# Chronic kidney disease impairs prognosis in electrical storm

**DOI:** 10.1007/s10840-020-00924-6

**Published:** 2021-01-23

**Authors:** Kathrin Weidner, Michael Behnes, Tobias Schupp, Jorge Hoppner, Uzair Ansari, Julian Mueller, Simon Lindner, Martin Borggrefe, Seung-hyun Kim, Aydin Huseyinov, Dominik Ellguth, Muharrem Akin, Dirk Große Meininghaus, Thomas Bertsch, Gabriel Taton, Armin Bollow, Thomas Reichelt, Niko Engelke, Linda Reiser, Ibrahim Akin

**Affiliations:** 1 First Department of Medicine, University Medical Centre Mannheim, Faculty of Medicine Mannheim, University of Heidelberg, Mannheim, 68167 Deutschland; 2 Clinic for Diagnostic and Interventional Radiology Heidelberg, University Heidelberg, Heidelberg, Germany; 3 Department of Cardiology and Angiology, Hannover Medical School, Hannover, Germany; 4 Department of Cardiology, Carl-Thiem-Klinikum Cottbus, Cottbus, Germany; 5 Institute of Clinical Chemistry, Laboratory Medicine and Transfusion Medicine, General Hospital Nuremberg, Paracelsus Medical University, Nuremberg, Germany

**Keywords:** Electrical storm, Chronic kidney disease, Long-term mortality, MACE

## Abstract

**Background:**

The study sought to assess the prognostic impact of chronic kidney disease (CKD) in patients with electrical storm (ES). ES represents a life-threatening heart rhythm disorder. In particular, CKD patients are at risk of suffering from ES. However, data regarding the prognostic impact of CKD on long-term mortality in ES patients is limited.

**Methods:**

All consecutive ES patients with an implantable cardioverter–defibrillator (ICD) were included retrospectively from 2002 to 2016. Patients with CKD (MDRD-GFR < 60 ml/min/1.73 m^2^) were compared to patients without CKD. The primary endpoint was all-cause mortality at 3 years. Secondary endpoints were in-hospital mortality, cardiac rehospitalization, recurrences of electrical storm (ES-R), and major adverse cardiac events (MACE) at 3 years.

**Results:**

A total of 70 consecutive ES patients were included. CKD was present in 43% of ES patients with a median glomerular filtration rate (GFR) of 43.3 ml/min/1.73 m^2^. CKD was associated with increased all-cause mortality at 3 years (63% vs. 20%; *p* = 0.001; HR = 4.293; 95% CI 1.874–9.836; *p* = 0.001) and MACE (57% vs. 30%; *p* = 0.025; HR = 3.597; 95% CI 1.679–7.708; *p* = 0.001). In contrast, first cardiac rehospitalization (43% vs. 45%; log-rank *p* = 0.889) and ES-R (30% vs. 20%; log-rank *p* = 0.334) were not affected by CKD. Even after multivariable adjustment, CKD was still associated with increased long-term mortality (HR = 2.397; 95% CI 1.012–5.697; *p* = 0.047), as well as with the secondary endpoint MACE (HR = 2.520; 95% CI 1.109–5.727; *p* = 0.027).

**Conclusions:**

In patients with ES, the presence of CKD was associated with increased long-term mortality and MACE.

## Introduction

Electrical storm (ES) is defined as ≥ 3 distinct episodes of sustained ventricular tachycardia (VT) or fibrillation (VF) within 24 h requiring implantable cardioverter–defibrillator (ICD) therapy [1–3]. The clinical presentation of ES is heterogeneous and differs between asymptomatic patients and those with severe hemodynamic instability or cardiac death [4]. ES is still associated with increased mortality of 40% at 1 year, whereas the causative pathology remains unclear in the vast majority of patients [3, 5]. Pathophysiologically, ES represents a condition associated with increased sympathetic activity [6, 7]. Several comorbidities coexisting in ES patients, such as congestive heart failure, metabolic syndrome, and chronic kidney disease (CKD), may further increase the sympathetic tone [6, 7]. In particular, CKD is a major burden in patients with cardiovascular diseases, whereas most studies usually exclude patients with advanced stages of CKD [8, 9]. Within the last decade, it has been demonstrated that even mild forms of CKD may be associated with cardiovascular morbidity and mortality [10]. Such patients are at increased risk of acute or chronic electrolyte alterations, autonomic imbalance, micro- and macroangiopathy, left ventricular hypertrophy or fibrosis and acquired QT prolongation. Alongside these risk factors, oxidative stress, metabolic acidosis, and hyperuremia increase the risk of ventricular tachyarrhythmias [10]. The imbalance of the autonomic nervous system, reflected by increased sympathetic activity, has been investigated within the causative context of ES [6, 11]. Consequently, a main objective in the therapy and prevention of ES represents  the reduction of sympathetic activity by administration of beta-blockers. Furthermore, unnecessary ICD shocks during ES should be avoided and alternative therapeutic options such as anti-tachycardia pacing (ATP) or attenuated VT detection cluster should be applied [2, 12]. It is important to identify clinical risk factors impacting the long-term prognosis of ES patients. However, the prognostic impact of CKD in patients suffering from ES has rarely been investigated. Therefore, the present study evaluates the long-term prognostic impact of CKD in patients with ES.

## Methods

### Study population

This retrospective study included all consecutive patients presenting with ES from 2002 until 2016 at one institution. ES was defined as ≥ 3 episodes of ventricular tachyarrhythmias delimited by at least 5 min leading to appropriate ICD therapy during a single 24-h time period [1]. Only ICD recipients were included.

All relevant clinical data were documented using the electronic hospital information system, ICD protocols, discharge letters, daily charts, patients’ files, and reports from diagnostic testing, including 12-lead electrocardiogram (ECG) and Holter ECG being assessed during clinical routine. In detail, data documentation comprised baseline characteristics, prior medical history, prior medical treatment, length of index stay, detailed findings of laboratory values at baseline, and data derived from all non-invasive or invasive cardiac diagnostics and device therapies, such as coronary angiography, electrophysiological examination, and imaging modalities, such as echocardiography or cardiac magnetic resonance imaging (cMRI). The documentation period lasted from the index event until 2016. Independent cardiologists performed documentation of all medical data at the time of the patients’ individual clinical presentation period, being blinded to final data analyses. The present study is derived from a retrospective analysis of the Registry of Malignant Arrhythmias and Sudden Cardiac Death–Influence of Diagnostics and Interventions (RACE-IT) and represents a single-center registry including consecutive patients presenting with ventricular tachyarrhythmias and aborted cardiac arrest being acutely admitted to the University Medical Center Mannheim (UMM), Germany (clinicaltrials.gov identifier NCT02982473) from 2002 until 2016. The registry was carried out according to the principles of the Declaration of Helsinki and was approved by the medical ethics committee II of the Faculty of Medicine Mannheim, University of Heidelberg, Germany.

### Risk stratification

For the present analysis, ES patients with CKD were compared to ES patients without CKD (non-CKD). Accordingly, CKD was defined as abnormalities of kidney function with health implications accompanied by a glomerular filtration rate (GFR) < 60 ml/min/1.73 m^2^ (GFR categories G3a–G5) and a duration > 3 months [13]. All CKD patients were included according to current guidelines, irrespective of the CKD stadium [13].

### Definition of endpoints

The primary endpoint was long-term all-cause mortality at 3 years. Secondary endpoints comprised in-hospital mortality, first cardiac rehospitalization, major adverse cardiac event (MACE), and ES-R at long-term follow-up of 3 years. The first cardiac rehospitalization was related to recurrent VT and VF, as well as related to cardiopulmonary resuscitation (CPR), acute heart failure, or acute myocardial infarction (AMI) [14]. AMI was defined according to current guidelines as the presence of an acute myocardial injury with clinical evidence of acute myocardial ischemia and detection of a rise and/or fall of cTn values with at least one value above the 99th percentile (URL). Furthermore, at least one of the following symptoms was present: symptoms of myocardial ischemia, new ischemic ECG changes, development of pathological Q waves, imaging evidence of new loss of viable myocardium or new regional wall motion abnormality in a pattern consistent with an ischemic etiology, and identification of a coronary thrombus by angiography [14, 15]. MACE were defined as the composite of AMI, target vessel revascularization (TVR) by percutaneous coronary intervention (PCI) or coronary artery bypass grafting (CABG), and the primary endpoint of all-cause mortality [16]. Recurrence of electrical storm (ES-R) was defined as the recurrence of further episodes of ES at follow-up beyond the initial 24 h of prior ES [2]. The follow-up period lasted until 2016. All-cause mortality was documented using our electronic hospital information system and by directly contacting state resident registration offices (bureau of mortality statistics) across Germany. Identification of patients was verified by place of name, surname, day and place of birth, and registered living address.

### Statistical methods

Quantitative data are presented as mean ± standard error of mean (SEM), median and interquartile range (IQR), and ranges depending on the distribution of the data and were compared using Student’s *t* -test for normally distributed data or the Mann–Whitney *U* test for non-parametric data. Deviations from a Gaussian distribution were tested by the Kolmogorov–Smirnov test. Spearman’s rank correlation for non-parametric data was used to test univariate correlations. Qualitative data are presented as absolute and relative frequencies and compared using the chi-squared test or Fisher’s exact test, as appropriate. The following analyses were applied stepwise to evaluate the prognostic value of predefined variables for all-cause mortality: Kaplan–Meier survival curves were calculated with log-rank testing for statistical significance. Univariable hazard ratios (HR) are given together with 95% confidence intervals. Multivariable Cox regression models with long-term mortality as the dependent variable were developed using the forward selection option, including variables of clinical prognostic relevance. The result of a statistical test was considered significant for *p* < 0.05, and a statistical trend was defined as *p* < 0.1. SAS release 9.4 (SAS Institute Inc., Cary, NC, USA) was used for statistics.

## Results

### Study population

A total of 70 patients with ES were included consecutively; 43% suffered from CKD (Table 1). The median creatinine level was 1.7 mg/dl (IQR 1–4 mg/dl) with a median GFR of 43.3 ml/min/1.73m^2^ and a median urea level of 71 mg/dl (IQR 58–93 mg/dl, data not shown). All patients showed a moderate functional impairment of the kidney at stages 3a and 3b, according to current guidelines [13]. None of the patients showed CKD stages 4 and 5. According to this, none of the patients was on hemodialysis [13]. As shown in Table 1, most patients were males (non-CKD 93% vs. CKD 80%; *p* = 0.122). CKD patients were older (71 vs. 65 years; *p* = 0.047) and showed higher rates of out-of-hospital CPR (1% vs. 20%; *p* = 0.015). Furthermore, significantly more CKD patients had left ventricular ejection fraction (LVEF) < 35% (86% vs. 53%; *p* = 0.015) accompanied by significantly higher rates of ischemic cardiomyopathy (40% vs. 87%; *p* = 0.001). Non-CKD patients showed a trend towards numerically higher aldosterone receptor antagonist therapy than CKD patients (31% vs. 13%, *p* = 0.089). No differences were found in potassium levels, beta-blocker, ACE/AT1 inhibitor, amiodarone, and diuretics. No differences were found for rates of AMI and atrial fibrillation (AF). Non-CKD patients showed a trend towards numerically higher rates of coronary angiography at index hospitalization (60% vs. 40%, *p* = 0.098). Accordingly, higher rates of coronary multivessel disease were found, whereas non-CKD patients showed numerically higher rates of coronary single-vessel disease or no evidence of CAD. No further differences were found regarding cardiovascular risk factors, comorbidities, electrocardiogram (ECG) data, ICD thresholds, and discharge medication (Table 1). Hemoglobin levels were significantly lower in the CKD group, reflecting the presence of renal anemia (median 12.2 vs. 14.3 g/dl).

**Table 1:** Baseline characteristics

Characteristic	Non-CKD (*n* = 40; 57%)		CKD (*n* = 30; 43%)		*p* value
Age, median (range)	65 (22–83)		71 (38–85)		*0.047*
Hemodialysis, *n* (%)	-	-	(0)	(0)	-
Male gender, *n* (%)	37	(93)	24	(80)	0.122
Cardiopulmonary resuscitation, *n* (%)					
Out-of-hospital	1	(1)	6	(20)	*0.015*
In-hospital	3	(8)	4	(13)	0.420
Cardiovascular risk factors, *n* (%)					
Arterial hypertension	21	(53)	22	(73)	0.076
Diabetes mellitus	7	(18)	9	(30)	0.258
Hyperlipidemia	13	(33)	14	(47)	0.228
Smoking	10	(25)	2	(7)	*0.044*
Cardiac family history	3	(8)	3	(10)	0.712
Comorbidities, *n* (%)					
Acute myocardial infarction	0	(0)	0	(0)	-
Atrial fibrillation	13	(33)	14	(47)	0.228
Liver cirrhosis	1	(3)	3	(10)	0.180
COPD	6	(15)	9	(30)	0.130
Prior stroke	5	(13)	8	(27)	0.131
Cardiomyopathy, *n* (%)					
Ischemic cardiomyopathy	16	(40)	26	(87)	*0.001*
Non-ischemic cardiomyopathy	4	(10)	3	(12)	1.000
Not documented	20	(50)	1	(1)	*0.001*
Channelopathies, *n* (%)					
Brugada syndrome	1	(3)	0	(0)	1.000
Long-QT syndrome	1	(3)	0	(0)	1.000
Short-QT syndrome	0	(0)	0	(0)	-
Coronary angiography, *n* (%)	24	(60)	12	(40)	0.098
Coronary one-vessel disease	5	(21)	0	(0)	0.405
Coronary two-vessel disease	3	(12)	2	(17)	
Coronary three-vessel disease	13	(54)	8	(67)	
Electrophysiological examination, *n* (%)	10	(25)	10	(33)	0.445
VT ablation	9	(23)	8	(27)	0.687
Laboratory data (mean ±SEM)					
Creatinine (mg/dl)	1.06 ± 0.003		1.49 ± 0.007		*0.001*
GFR (ml/min/1.73 m^2^)	60.6 ± 0.53		43 ± 3.73		*0.001*
Hemoglobin (g/dl)	13.8 ± 0.3		12.2 ± 0.4		*0.001*
Potassium (mmol/l)	4.0 ± 0.1		4.2 ± 0.1		0.300
C-reactive protein (mg/dl)	15.1 ± 3.9		49.9 ± 12.1		*0.003*
Troponin I (μg/l)	0.3 ± 0.1		0.4 ± 0.1		0.549
Medication at discharge, *n* (%)					
Beta-blocker	38	(95)	30	(100)	0.230
ACE inhibitor/ARB	34	(85)	21	(75)	0.302
Statin	23	(58)	14	(50)	0.541
Amiodarone	24	(60)	18	(64)	0.720
Aldosterone receptor antagonist	12	(31)	4	(13)	0.089
Diuretics	23	(59)	20	(67)	0.513
ECG data (mean ± SEM)					
PQ	226 ± 16		204 ± 15		0.376
QRS	128 ± 21		127 ± 16		0.957
QT	450 ± 18		423 ± 23		0.378
LVEF, *n* (%)					
≥ 55%	7	(19)	0	(0)	*0.015*
54–45	4	(11)	3	(11)	
44–35%	6	(17)	1	(3)	
< 35%	19	(53)	24	(86)	
Type of ICD, *n* (%)					
ICD	36	(90)	26	(87)	0.233
CRT-D	2	(5)	4	(13)	
s-ICD	2	(5)	0	(0)	
ICD indication, *n* (%)					
Primary prevention	15	(38)	11	(38)	0.971
Secondary prevention	25	(62)	18	(62)	
ICD programming, bpm, median (IQR)					
VT detection threshold	171 (158–176)		167 (154–171)		0.561
VF detection threshold	214 (214–221)		214 (214–222)		0.761

*ACE* angiotensin-converting enzyme, *AKI* acute kidney injury *ARB* angiotensin receptor blocker, *COPD* chronic obstructive pulmonary disease, *CKD* chronic kidney disease, *CPR* cardiopulmonary resuscitation, *CRT-D* cardiac resynchronization therapy defibrillator, *ECG* electrocardiogram, *ES* electrical storming, *GFR* glomerular filtration rate, *ICD* implantable cardioverter–defibrillator, *IQR* interquartile range, *LVEF* left ventricular ejection fraction, *SEM* standard error of measurement, *VF* ventricular fibrillation, *VT* ventricular tachycardia

Italic indicates the significance level *p* < 0.05

Most ES patients had an ICD implanted for secondary prevention (62% vs. 38%). The most common ICD type was the conventional ICD (90% vs. 87%), followed by the cardiac resynchronization therapy defibrillator (CRT-D). Notably, CKD patients had numerically higher rates of CRT-D (13% vs. 5%) and no s-ICD implanted (0% vs. 5%) (Table 1).

### Primary endpoint all-cause mortality

**Fig 1:**
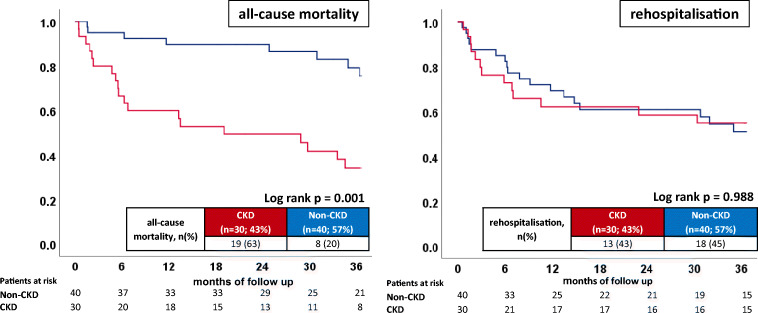
Prognostic impact of CKD on long-term all-cause mortality (left panel) and rehospitalization (right panel) in patients presenting with ES

All patients were followed up regarding the primary endpoint of all-cause mortality at 3 years (median 2.45 years; IQR 1.01–4.77 years). CKD patients were associated with increased rates of long-term all-cause mortality (63% vs. 20%; log-rank *p* = 0.001; Table 2; Fig. 1 left panel) compared with non-CKD patients. Accordingly, the risk of all-cause mortality was higher in CKD patients (HR = 4.293; 95% CI 1.874–9.836; *p* = 0.001).

**Table 2:** Primary and secondary endpoints

Characteristic	Non-CKD (*n* = 40; 57%)		CKD (*n* = 30; 43%)		*p* value
Primary endpoint, *n* (%)					
All-cause mortality at 3 years	8	(20)	19	(63)	*0.001*
Secondary endpoints, *n* (%)					
In-hospital mortality	0	(0)	2	(7)	0.180
First cardiac rehospitalization	18	(45)	13	(43)	0.889
MACE	12	(30)	17	(57)	*0.025*
ES recurrence	8	(20)	9	(30)	0.334

*ES* electrical storm, *MACE* major adverse cardiac events

Italic indicates the significance level *p* < 0.05

### Secondary endpoints

**Fig 2:**
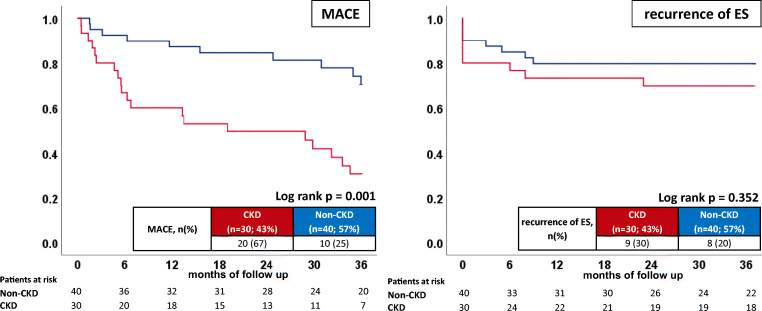
Prognostic impact of CKD on MACE (left panel) and recurrences of ES (right panel) in patients presenting with ES

In contrast, in-hospital mortality rates were similar in both groups (7% vs. 0%; log-rank *p* = 0.180). Furthermore, rates of first cardiac rehospitalization (45% vs. 43%; log-rank *p* = 0.988) were similar in both groups (Table 2; Fig. 1 right panel). No differences were seen for ES-R (30% vs. 20%; *p* = 0.352). Additionally, increased rates of MACE were seen in CKD patients (57% vs. 30%; *p* = 0.025; HR 3.597; 95% CI 1.679–7.708, *p* = 0.001; Table 2; Fig. 2 left panel).

### Multivariable Cox models

In multivariable Cox regression analyses, the presence of CKD was still associated with increased long-term all-cause mortality (HR = 2.397; 95% CI 1.012–5.697; *p* = 0.047) besides LVEF < 35% (HR 9.015, 95% CI 1.141–71.126, *p* = 0.037) (Table 3). The presence of CKD was also associated with the secondary endpoint MACE (HR = 2.520; 95% CI 1.109–5.727; *p* = 0.027) (Table 4).

**Table 3:** Multivariable Cox regression model to evaluate prognostic factors associated with long-term mortality at 3 years

Variable	HR	95% CI	*p* value
Age	1.023	0.978–1.071	0.316
Diabetes mellitus	0.896	0.385–2.087	0.799
Chronic kidney disease^a^	2.397	1.012–5.697	*0.047*
LVEF < 35%	9.015	1.141–71.126	*0.037*
Atrial fibrillation	1.338	0.617–2.905	0.461

*CI* confidence interval, *HR*, hazard ratio, *LVEF* left ventricular ejection fraction

^a^Defined as creatinine > 1.2 mg/dl

Italic indicates the significance level *p* < 0.05

**Table 4:** Multivariable Cox regression model to evaluate prognostic factors influencing the secondary endpoint MACE

Variable	HR	95% CI	*p* value
Age	1.013	0.975–1.052	0.496
Diabetes mellitus	1.010	0.451–2.263	0.981
Chronic kidney disease^a^	2.520	1.109–5.727	*0.027*
LVEF < 35%	2.192	0.679–7.073	0.189
Atrial fibrillation	1.363	0.650–2.859	0.412

*CI* confidence interval, *HR* hazard ratio, *LVEF* left ventricular ejection fraction

^a^Defined as creatinine > 1.2 mg/dl

Italic indicates the significance level *p* < 0.05

## Discussion

The present study evaluates the prognostic impact of CKD in consecutive high-risk patients presenting with ES on admission. This data suggests that ES patients reveal a higher long-term mortality at 3 years in the presence of CKD. Respectively, increasing rates of the secondary endpoint MACE were seen in CKD patients. In contrast in-hospital mortality rates, risk of first cardiac rehospitalization, and ES-R were not affected by CKD. Prognostic differences were demonstrated even within the multivariable Cox regression model, where both CKD and LVEF < 35% were still associated with long-term mortality at 3 years. This study identifies the presence of CKD as a robust predictor of adverse prognosis in ES patients.

CKD is an independent predictor of cardiovascular morbidity and mortality [17, 18]. The most common cause of death in hemodialysis patients is sudden cardiac death (SCD) [10]. Although dialysis patients show the highest risk of cardiovascular events, even mild CKD stages are associated with ventricular tachyarrhythmias and SCD [18].

Potential risk factors and the resulting preventive therapeutic options of patients with ventricular tachyarrhythmias have been discussed continuously within the past years [12, 19]. According to international guideline recommendations, the implantation of an ICD is strongly recommended in patients with systolic heart failure defined as LVEF < 35% irrespective of the underlying cardiac disease [20]. However, especially in the presence of ischemic cardiomyopathy, as defined by a history of relevant CAD or prior myocardial infarction, ICD implantation was shown to be associated with reduced all-cause mortality [19]. Although ICD implantation is recommended for patients with systolic heart failure and non-ischemic (dilatative) cardiomyopathy (DCM) [20], the DANISH trial recently reported that primary preventive ICD implantation in patients with non-ischemic cardiomyopathy was not associated with improved long-term prognosis (i.e., death from any cause and cardiovascular death). However, the risk of sudden cardiac death was significantly reduced in the ICD group [12]. We recently demonstrated that neither the presence of ischemic cardiomyopathy compared to non-ischemic cardiomyopathy revealed any differences in prognostic outcomes in ICD recipients presenting with ventricular tachyarrhythmias at index. However, patients with non-ischemic cardiomyopathy showed higher rates of recurrent ventricular tachyarrhythmias at 1 year [21].

Focusing on the prognostic impact of CKD in patients with ventricular tachyarrhythmias—representing a not well-studied risk factor in this subset of patients—it was demonstrated that CKD patients were significantly associated with increased long-term mortality at 2 years, cardiac death, and in-hospital death, which is also reflected within the corresponding RACE-IT CKD risk score [22]. Even in ICD recipients, only CKD was still associated with increased long-term mortality, recurrent ventricular tachyarrhythmias, and appropriate device therapies at 5 years [23].

In clear contrast, data in ES patients with CKD is rare. Potential risk factors for ES are widely discussed, and the specific causative pathology for the development of ES is not yet fully understood, not even in higher-risk patients with relevant comorbidities, such as CKD and heart failure [3, 5]. As demonstrated by the present analysis, the presence of both CKD and LVEF < 35% was shown to be significantly associated with increased all-cause mortality. Both comorbidities may reflect the presence of the cardiorenal syndrome, which is defined as “disorders of the heart and kidneys, whereby acute or chronic dysfunction in one organ may induce acute or chronic dysfunction of the other” [8, 24]. The cardiorenal syndrome is categorized into 5 subtypes based on the organ presumed to be the primary trigger [8, 24]. Although this definition is of clinical importance, it reveals less information about the underlying pathophysiological pathways. Interstitial fibrosis in the heart, vessel wall structure, and kidney has been identified as responsible pathogenetic factors in most types of the cardiorenal syndrome [25]. In the present analysis, CKD patients with ES were older and had a numerically increased cardiovascular risk profile. Aging and risk factors, such as arterial hypertension, dyslipidemia, and diabetes mellitus, may lead to sympathetic neurohumoral activation, chronic inflammation, and oxidative stress related to endothelial dysfunction. In turn, these conditions may alleviate, leading to heart and kidney failure due to the increasing amount of interstitial fibrosis and activation of the renin–angiotensin–aldosterone system (RAAS). Myocardial fibrosis attenuates cardiomyocyte coupling and thereby may enhance arrhythmogenicity [10]. Therefore, interstitial fibrosis may reflect the main causative pathology in cardiorenal syndrome [24, 25].

Furthermore, other explanations for the increased cardiovascular mortality in CKD patients do exist [10]. Myocardial ischemia related to coronary artery disease represents major comorbidity in CKD patients [10]. It has recently been demonstrated that ventricular tachyarrhythmias and SCD after myocardial infarction are more common in the presence of CKD [26]. In the present study, patients with CKD showed significantly higher rates of ischemic cardiomyopathy, which might further confirm these findings and explain the increased long-term mortality and higher rates of MACE in the present study evaluating patients with ES.

The present study demonstrated that CKD patients presenting with ES were associated with increased rates of long-term mortality and MACE compared to non-CKD patients. This negative prognostic impact of CKD in ES patients may be related to the cardiorenal syndrome due to myocardial and renal fibrosis, increased cardiovascular risk profile, and ischemic cardiomyopathy, which may explain the adverse prognostic impact of CKD in ES patients. However, further–at best-randomized controlled trials are needed to investigate the prognostic impact, its pathophysiological pathways, and potential therapeutic options in high-risk ES patients with CKD. The supposed benefit of excluding CKD patients from RCT, especially in high-risk patients with ES, needs to be further debated [1, 8].

## Study limitations

The present study is based on rather small sample size, with only 70 patients included in a retrospective and observational single-center registry. Rehospitalization rates were only documented within our own institution. Patients with prolonged hemodynamic instability and lethal outcome before admission and those not surviving out-of-hospital CPR without transfer to the heart center were not included in this study. Ablation rates among ES patients were low, possibly preventing showing a beneficial effect of ablation. Future prospective randomized controlled trials are needed to further clarify the prognostic impact of VT ablation in ES patients.

## Conclusion

In patients with ES, the presence of CKD is significantly associated with increased rates of long-term mortality at 3 years and MACE compared with non-CKD patients.
